# Dose–response study of prophylactic nitroglycerin for prevention of pituitrin-induced hypertension during laparoscopic myomectomy: a prospective, randomized study

**DOI:** 10.3389/fmed.2023.1186041

**Published:** 2023-07-03

**Authors:** Jin Wang, Qiang Xu, Fei Xiao, Gang Chen

**Affiliations:** ^1^Department of Anesthesia, Sir Run Run Shaw Hospital, Zhejiang University School of Medicine, Hangzhou, Zhejiang, China; ^2^Department of Anesthesia, Jiaxing University Affiliated Women and Children Hospital, Jiaxing, Zhejiang, China

**Keywords:** pituitrin, nitroglycerin, laparoscopy, leiomyoma, hypertension

## Abstract

**Objective:**

To determine the dose–response of nitroglycerin in preventing pituitrin-induced hypertension in patients undergoing laparoscopic myomectomy.

**Methods:**

Hundred patients scheduled for elective laparoscopic myomectomy were randomly allocated into one of five groups (*n* = 20) to receive intravenous bolus of prophylactic nitroglycerin at 0, 50, 75, 100, and 125 μg one minute following administration of 3 IU of pituitrin into the myometrium. The patients were monitored for pituitrin-induced hypertension with the primary outcome to determine the effective dose of prophylactic nitroglycerin, defined as complete prevention of pituitrin-induced hypertension during the study period. Probit analysis was used to calculate the median effective dose (ED_50_) and 95% effective dose (ED_95_) of prophylactic nitroglycerin.

**Results:**

Hypertension occurred in 19/20, 10/20, 8/20, 2/20, and 1/20 in patients who received 0, 50, 75, 100, and 125 ug of prophylactic nitroglycerin, respectively. The calculated ED_50_ and ED_95_ of nitroglycerin for preventing hypertension were 54 μg (95%CI: 35~66 μg) and 136 μg (95%CI: 105~289 μg).

**Conclusion:**

A prophylactic bolus of nitroglycerin administered immediately following injection of pituitrin into the myometrium during laparoscopic myomectomy effectively prevented pituitrin-induced hypertension, with the ED_50_ and ED_95_ of 54 μg and 136 μg, respectively. This information would be useful for clinical practice.

**Clinical trial registration:**

www.chictr.org.cn, Identifier ChiCTR2200062282.

## Introduction

To decrease intraoperative bleeding, shorten operative time, and reduce surgical complications, vasopressin is commonly used as an injection into the myometrium during laparoscopic myomectomy (1, 2). However, synthetic vasopressin is not available in some parts of the world including China. Pituitrin, a bovine posterior pituitary extract containing vasopressin and oxytocin, has been considered as a well-accepted alternative to vasopressin during myomectomy owing to its similar clinical effect as that of synthetic vasopressin and its lower price (3–5). However, pituitrin can cause drastic fluctuations in hemodynamics, including significantly increasing blood pressure, and slowing the heart rate, which can induce cardiovascular and cerebrovascular events (6–8). Prior studies have shown that intravenous nitroglycerin can decrease the incidence of hypertension and stabilize the hemodynamics (4). However, the optimal dose of nitroglycerin for the prevention of pituitrin-induced hypertension during laparoscopic myomectomy has been not determined.

In this study, the probit regression method was used to calculate the median effective dose (ED50) and 95% effective dose (ED95) of nitroglycerin given as a prophylactic bolus during laparoscopic myomectomy to prevent pituitrin-induced hypertension.

## Materials and methods

### Participants

This study was approved by the Ethics Committee of Jiaxing University Affiliated Women and Children’s Hospital (batch no. 2022027). Written informed consent was obtained from all subjects involved in the trial. The study was performed in accordance with the principles stated in the Declaration of Helsinki.

From August 2022 to October 2022, 100 ASA I~II patients, aged 26–60 years, scheduled to undergo laparoscopic myomectomy in our hospital were enrolled in this study. Exclusion criteria were obesity [body mass index (BMI) > 35 kg/m^2^], pre-existing hypertension (SBP > 140 mm Hg), prior occurrence of hypotension at the time of nitroglycerin administration, diabetes, heart disease and other internal diseases, endocrine and central nervous system diseases, diagnosis or tendency of having angle-closure glaucoma, >5 principal myomas or maximal diameter > 10 cm, nitroglycerin contraindications, and refusal to participate in the trial. According to a computer-generated random number sheet, patients were randomly allocated into 5 groups and received 0, 50, 75, 100, 125 μg nitroglycerin, respectively.

After entering the operating room, ECG, HR, BP and SpO_2_ were routinely measured and recorded for all patients. The depth of anesthesia was monitored by entropy index. Continuous invasive blood pressure monitoring was performed by left radial artery catheterization, and, after a brief resting period, baseline arterial blood pressure and heart rate (HR) were determined by calculating the mean of 3 consecutive measurements of systolic blood pressure (SBP) and HR at 3-min intervals. Under total intravenous anesthesia with endotracheal intubation, propofol (effector compartment concentration 3 μg/mL) and remifentanil (effector compartment concentration 3 ng/mL) were infused. After the patient lost consciousness, cisatracurium 0.15 mg/kg and sufentanil 0.2 μg/kg were intravenously injected. Mechanical ventilation was established using IPPV with a tidal volume of 6–8 mL/kg and a frequency of 12–16 breaths/min, maintaining an end-expiratory carbon dioxide between 35 and 45 mmHg. In the maintenance stage of anesthesia, targeted infusion of propofol and remifentanil were adjusted to effector concentrations of 3–5 μg/mL and 3–6 ng/mL, respectively, and the entropy index was maintained at 40–60 (9).

The laparoscopic procedure was performed consistently by the same surgical team. The patient was placed in a 30° reverse Trendelenburg lithotomy position, and the pneumoperitoneum pressure was maintained between 10 and 15 mmHg. During surgery, 3 IU of pituitrin (Anhui Hongye Pharmaceutical Co., LTD., batch number: 201203) were diluted with 0.9% normal saline to a total volume of 10 mL, then injected into the myometrium surrounding the target leiomyoma under laparoscopic surveillance. One minute following injection of pituitrin, an intravenous bolus of nitroglycerin (Shandong Shenglu pharmaceutical co., LTD., batch number: 2007251) was given, according to the dose appropriate for each study group (0, 50, 75, 100, and 125 μg). The drugs in each group were prepared by the same anesthesiologist who was not involved in patient care or data collection, and the surgeons and anesthesiologists in the operating room were blinded to the dose contained in the solution.

Hypertension was defined as an increase in SBP > 120% of baseline, or the value of SBP > 160 mmHg, and/or DBP > 90 mmHg; such hypertension would prompt treatment with a bolus of 50 μg intravenous nitroglycerin. Hypotension was defined as a decrease in SBP > 20% of baseline or a value < 90 mm Hg. If hypotension was accompanied by tachycardia (HR > 100 beats per minute), it was treated with a bolus of 50 μg intravenous phenylephrine; if hypotension was accompanied by bradycardia (HR < 50 beats per minute), it was treated with a 6 mg bolus of intravenous ephedrine. Each occurrence of the above measures was considered as an intraoperative intervention.

### Measurements

Demographic characteristics such as age, height and weight were recorded as well as baseline BP and surgical parameters, including the number and size of tumors, duration of surgery and blood loss. Physician interventions (intravenous nitroglycerin, phenylephrine, ephedrine or atropine) for treating hypertension, hypotension or bradycardia were also recorded as well as the details of the adverse events.

### Sample size estimation

Sample size was calculated with the Cochran-Armitage test using PASS (version 15.0.5; NCSS, LLC, Kaysville, Utah, United States). Calculations were based on early preliminary data of four groups with nitroglycerin injection doses of 50, 75, 100, and 125 μg, and the corresponding proportions of hypertension were 60% (6/10), 40% (4/10),10% (1/10), and 10% (1/10), respectively. We determined that a total sample size of 67 patients (17 per group) would have a 90% power to detect a linear trend in the proportion of patients with hypertension among groups by using a Z test with continuity correction and a significance level of 0.05. Taking into account the 10% dropout rate in each group and the addition of a control group in this study, the sample size was increased to 100 patients.

### Statistical analysis

We assessed whether continuous variables were normally distributed using the Kolmogorov–Smirnov test. Data with a normal distribution were presented as mean ± SD and analyzed via one-way analysis, and the post-hoc Bonferroni test was used for pairwise comparisons. Data that showed a nonnormal distribution, such as the times of interventions, were presented as median (range) and tested with the Kruskal–Wallis test, and the post-Dunn’s test was applied to analyze pairwise comparisons. Categorical trend data such as the incidence of hypertension were analyzed using the Cochran-Armitage χ^2^ test for trend. If the overall test of difference among groups was significant, chi-squared tests were used for pairwise comparisons. Variations in SBP within the first 30 min after pituitrin injection were measured using a two-factor repeated measurement ANOVA, and post-event comparisons were performed using a Bonferroni test. The ED50 and ED95 for an effective prophylactic nitroglycerin infusion dose were determined using Probit regression. Analyses were performed using IBM SPSS Statistics for Windows version 23.0 (IBM Corp, Armonk, New York, United States) and GraphPad Prism version 9.1.2 (GraphPad Software Inc., San Diego, California, United States). *p*-values < 0.05 were considered statistically significant (two-sided). For multiple comparisons, corrected significance levels were used (Bonferroni correction).

## Results

**Figure 1:**
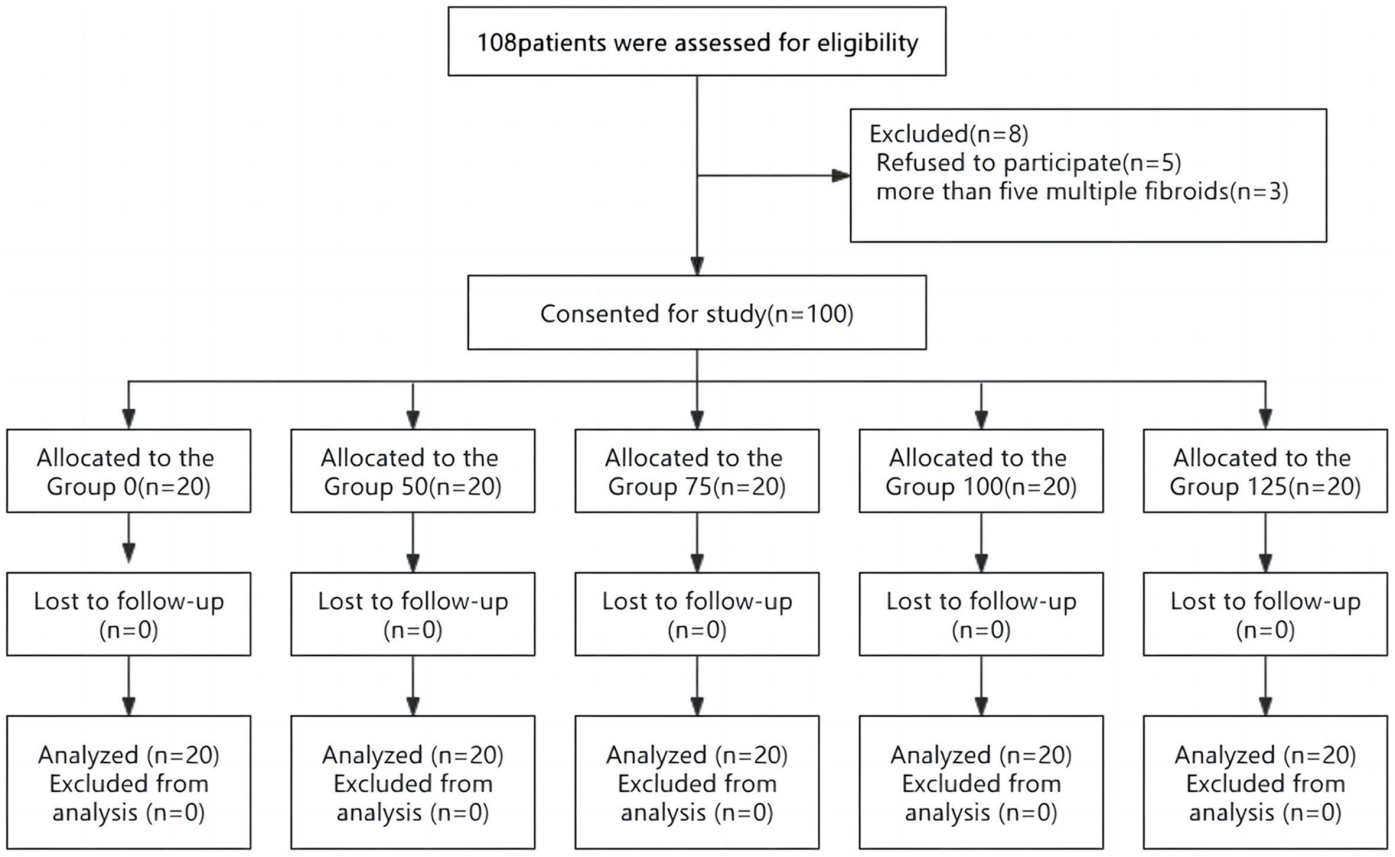
Consolidated standards of reporting trials diagram showing patient recruitment and flow.

Initially, 108 patients undergoing laparoscopic myomectomy were enrolled and checked for eligibility. Five patients declined to take part in this clinical trial, three patients were excluded from the analysis because more than five fibroids were found intraoperatively (Figure 1). No intergroup differences were noted in demographic data, basal blood pressure, the size and number of tumors, surgical times, and blood loss (Table 1).

**Table 1:** Demographic characteristics and surgical parameters.

Characteristic	Group 0 (n = 20)	Group 50 ( n = 20)	Group 75 ( n = 20)	Group 100 ( n = 20)	Group 125 ( n = 20)
Age/years	44.3 ± 4.7	42.9 ± 5.6	43.7 ± 6.1	43.4 ± 4.6	41.6 ± 6.5
Height/cm	160.0 ± 4.5	159.9 ± 4.7	157.6 ± 5.3	158.9 ± 4.8	158.7 ± 5.6
Weight/kg	61.5 ± 7.5	57.2 ± 5.6	59.1 ± 7.0	55.9 ± 7.0	57.4 ± 6.9
Basal SBP/mmHg	121.9 ± 8.8	123.8 ± 8.5	122.3 ± 6.9	121.2 ± 9.9	126.0 ± 10.5
Basal DBP/mmHg	75.4 ± 7.7	77.0 ± 9.5	72.9 ± 5.5	75.7 ± 9.2	73.8 ± 9.0
Number of tumors	1.0 (1–4)	1.0 (1–5)	1.0 (1–4)	1.0 (1–4)	1.5 (1–4)
Tumor diameter/cm	5.2 ± 1.7	4.9 ± 1.5	5.7 ± 1.4	5.6 ± 1.7	6.0 ± 1.5
Operation time/min	1.3.1 ± 29.4	90.6 ± 21.4	83.6 ± 24.2	90.6 ± 21.1	97.8 ± 31.3
Blood loss/mL	80 (30–200)	100 (20–200)	55 (50–200)	50 (20–150)	80 (30–200)

Data are presented as mean ± SD or median (range).

Hypertension occurred in 19/20, 10/20, 8/20, 2/20, and 1/20 in patients who received 0, 50, 75, 100, and 125 μg of prophylactic nitroglycerin, respectively. There were significant intergroup differences in the incidence of hypertension (Table 2, P < 0.001).

**Table 2:** Cardiovascular responses and interventions.

Outcome	Group 0 ( n = 20)	Group 50 ( n = 20)	Group 75 ( n = 20)	Group 100 ( n = 20)	Group 125 ( n = 20)	P -value
Hypertension	19 (95)^a^	10 (50)^b^	8 (40)	2 (10)	1 (5)	<0.001
Hypotension	0 (0)	0 (0)	0 (0)	0 (0)	3 (15)	0.064
Bradycardia	8 (40)^c^	5 (25)^e^	2 (10)	0 (0)	0 (0)	<0.001
Tachycardia	0 (0)	0 (0)	0 (0)	0 (0)	0 (0)	—
interventions	2 (0–4)^d^	1 (0–3)^e^	0 (0–3)^f^	0 (0–2)	0 (0–1)	<0.001

Data are presented as *n* (%) or median (range). Categorical data were analyzed using the Cochran–Armitage χ2 test for trend. Bonferroni corrected significance level is 0.05/10 = 0.005. Times of interventions, were tested with the Kruskal–Wallis test, and the post-Dunn’s test was applied to analyze pairwise comparisons.

a P = 0.001 vs. group 50, P < 0.001 vs. group 75, 100, and 125, respectively.

b P = 0.001 vs. group 125.

c P = 0.002 vs. group 100 and 125, respectively.

d P = 0.006 vs. group 50, P < 0.001 vs. group 75, 100, and 125, respectively.

e P = 0.009 vs. group 100, P = 0.005 vs. group 125.

f P = 0.009 vs. group 125.

**Figure 2:**
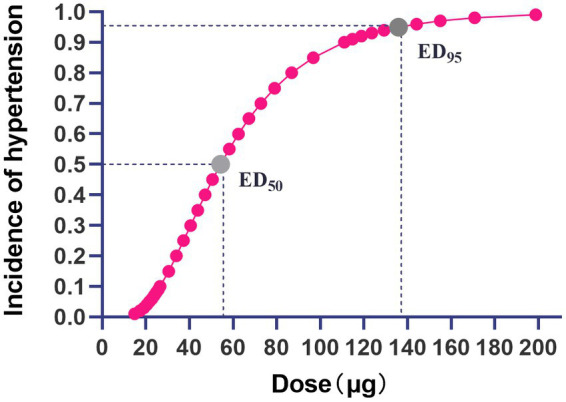
Dose–response curve of nitroglycerin for prevention of pituitrin-induced hypertension during laparoscopic myomectomy. The gray dots on the curve represent the ED_50_ and ED_95_ values of nitroglycerin for preventing pituitrin-induced hypertension, and the values were 54 μg (95%CI:35~66 μg) and 136 μg (95%CI: 105~289 μg), respectively.

The dose–response curve of nitroglycerin for preventing pituitrin-induced hypertension during laparoscopic myomectomy is presented in Figure 2. The ED_50_ and ED_95_ values were 54 μg (95%CI: 35–66 μg) and 136 μg (95%CI: 105–289 μg), respectively.

Variations of SBP within 30 min after injection of pituitrin for the five groups are presented in Table 3. Analysis showed that SBP over time was significantly different within each group (*p* < 0.001). Compared with 2 min before injection, SBP of 0 μg group was increased at 2 to 30 min (*p* < 0.05), SBP of 50 and 100 μg group increased at 3 to 10 min (*p* < 0.05), the SBP of 75 μg group increased from 2 to 10 min (*p* < 0.05), SBP of 125 μg group was increased at 10 min (*p* < 0.05). Comparison between groups at 3 and 5 min showed that the differences were statistically significant (*p* < 0.001). Compared with 0 μg group, SBP of 50, 75, 100, and 125 μg groups were decreased (*p* < 0.05).

**Table 3:** Variations of SBP within 30 min after injection of pituitrin.

Time	Group 0 (*n* = 20)	Group 50 (*n* = 20)	Group 75 (n = 20)	Group 100 (*n* = 20)	Group 125 (*n* = 20)	*F*-value	*P-*value
−2 min	119.50 ± 9.70	120.60 ± 11.64	114.25 ± 8.88	115.00 ± 12.40	118.60 ± 13.83	1.208	0.312
0 min	114.80 ± 14.12	116.75 ± 15.12	112.35 ± 17.03	110.00 ± 12.12	111.75 ± 14.92	0.648	0.629
1 min	119.80 ± 14.16	120.50 ± 14.99	115.25 ± 18.07	114.70 ± 10.96	118.40 ± 12.85	0.669	0.615
2 min	133.55 ± 15.45^a^	131.75 ± 18.59	127.85 ± 18.89^a^	126.20 ± 17.00	118.95 ± 15.81	2.186	0.076
3 min	150.55 ± 13.30^a^	132.70 ± 18.92^ab^	130.60 ± 16.56^ab^	127.40 ± 14.67^ab^	124.80 ± 12.71^b^	8.690	<0.001
5 min	147.20 ± 15.95^a^	132.15 ± 15.96^ab^	130.45 ± 16.61^ab^	127.25 ± 16.01^ab^	126.85 ± 13.60^b^	5.669	<0.001
10 min	137.45 ± 14.92^a^	134.85 ± 15.08^a^	129.15 ± 14.94^a^	127.50 ± 16.37^a^	128.85 ± 14.26^a^	1.642	0.170
30 min	129.75 ± 10.69^a^	128.50 ± 16.18	121.65 ± 14.68	120.80 ± 16.79	120.10 ± 14.33	1.950	0.108
*F-*value	21.645	5.531	6.699	5.520	5.564		
*P-*value	<0.001	<0.001	<0.001	<0.001	<0.001		

Data are presented as mean ± SD, and measured using a two-factor repeated measurement ANOVA, and post-event comparisons were performed using a Bonferroni test.

a *p* < 0.05 vs. −2 min; ^b^*p* < 0.05 vs 0 μg.

The median number of physician interventions in the 0, 50, 75, 100, and 125ug groups were 2 (0–4), 1 (0–3), 0 (0–3), 0 (0–2), and 0 (0–1), respectively, and there were statistically significant differences in the number of required interventions among the groups (Table 2, P < 0.001).

The incidences of intraoperative hypotension, bradycardia and tachycardia are presented in Table 2. The incidence of hypertension decreased with increasing nitroglycerin dose and reactive hypotension occurred in 3 patients in group 125, with an incidence of 15%, but there was no significant difference compared with other groups ( P = 0.064).

## Discussion

In this prospective, randomized, double-blind study, we determined the dose–response of nitroglycerin in preventing pituitrin-induced hypertension in patients undergoing laparoscopic myomectomy; the ED_50_ and ED_95_ values were 54 μg (95%CI: 35–66 μg) and 136 μg (95%CI: 105~289 μg), respectively.

After injection of pituitrin into the myometrium surrounding the target leiomyoma, hemodynamics showed a biphasic pattern—a transient decrease in blood pressure and a reflex increase in heart rate, followed by a continuous and dramatic increase in blood pressure with a slowing of heart rate. It is critical to prevent these hemodynamic changes from resulting in cardiovascular or cerebrovascular accidents, particularly in a patient with potentially a fragile cardiovascular or cerebrovascular status. In this study, we calculated the ED_50_ and ED_95_ of prophylactic bolus IV nitroglycerin in preventing hypertension induced by pituitrin during laparoscopic myomectomy by grouping dosimetry and probit regression analysis. To our knowledge, this is the first study to investigate the dose–response relationship of nitroglycerin in prevention of pituitrin-induced hypertension; the results could provide valuable clinical information of the pharmacodynamics of nitroglycerin.

Pituitrin contains two active components, oxytocin and vasopressin (10). Oxytocin acts as a prompt, direct peripheral vasodilator, resulting in decreased blood pressure and a reflexively increased heart rate. However, this process is only transient (11, 12). Then the vasopressin effect becomes apparent, with peripheral vasoconstriction, resulting in sharply increased blood pressure (13, 14). These changes can set off a chain reaction of events, with hypertension activating baroreceptors, increasing the efferent impulse of the vagus nerve, weakening the efferent impulse of the cardiac sympathetic nerves, slowing the heart rate, reducing the contractility of the myocardium, even leading to cardiac arrest in serious cases. Simultaneously, vasopressin causes coronary artery vasoconstriction with reduced coronary blood flow (15, 16). Previous studies have suggested that the total dose of pituitrin should not exceed 6 international unit (IU), and the concentration should not exceed 0.3 IU/mL, otherwise severe hemodynamic fluctuations could result (1, 6, 8).

Nitroglycerin, which exerts its vasodilator action via the NO-cGMP pathway, is one of the most used antihypertensive drugs in clinical practice (17). Previous studies have shown that intravenous injection of nitroglycerin could offset the side effects of using pituitrin during laparoscopic myomectomy (4). However, the optimal dose of nitroglycerin had not been reported, therefore, the impetus to conduct the present study.

Comparing the five different doses of prophylactic nitroglycerin (0, 50, 75, 100, and 125 μg) to prevent pituitrin-induced hypertension during laparoscopic myomectomy, the results demonstrated that the incidence of pituitrin-induced hypertension significantly decreased as the intravenous nitroglycerin bolus dose increased. However, the dose escalation was also associated with a corresponding increase in the incidence of hypotension, although not statistically significant. Therefore, anesthesiologists must take care to balance these factors when determining the appropriate dose in their clinical practice.

During this study, great caution was used in administering nitroglycerin as a routine measure to prevent pituitary-induced hypertension, recognizing that it could result in unexpected hypotension in some cases, as patient’s response to nitroglycerin could be quite variable. With respect to this important situation, we provided full dose–response information (from ED1 to ED99) of prophylactic nitroglycerin for clinical reference. Specifically, close monitoring of the patient’s response was assured in order to treat hypotension or hypertension immediately after using these vasoactive agents.

To identify the optimal dose of prophylactic nitroglycerin to prevent pituitrin-induced hypertension, our results determined that to protect 95% of the patients from experiencing pituitrin-induced hypertension, the prophylactic dose would be 136 μg (95%CI, 105~289 μg). However, because 3 (15%) patients experienced hypotension in the 125 μg group, one could logically be concerned that at a higher dose of 136 μg, an increased frequency of hypotension would be incurred. Moreover, there was no significant difference between the incidence of hypertension in group 100 and group 125, and no patient experienced hypotension in group 100. Therefore, we would favor 100 μg as an initial dose to prevent the hypertension, with adjustments to the dosage according to variations in the blood pressure. In the pretest, we observed a biphasic change in blood pressure, with an initial decrease after injection followed by an increase, consistent with the results of Guo et al. (4). We set the administration time at 1 min following the pituitrin injection to avoid aggravating the blood pressure reduction caused by oxytocin. The hypotension episodes observed in the present study were transient and did not require pharmacological treatment. Notably, the incidence of bradycardia in groups 75, 100, and 125 was significantly lower than in groups 0 and 50, and there was no tachycardia in any of the groups.

Furthermore, to maintain smooth, consistent hemodynamics during the operative procedure, the results showed that the high dose group was associated with fewer physician interventions, compared with the low dose groups. This further supported 100 μg nitroglycerin as an initial dose, to maintain a stable hemodynamic state, and reduce the need for physician interventions.

There are a number of limitations in this study. First, due to the strict inclusion and exclusion criteria, some patients were excluded, so generalizability of the results might be limited. Second, if different doses of pituitrin would be administered, the prophylactic nitroglycerin dose would likely need to be adjusted as well. Finally, the sample was calculated according to the dose–response relationship for bolus nitroglycerin, which was the primary objective of this study for which the study was powered. Secondary outcomes, however, may not be sufficiently powered for statistical analysis and statistical errors may exist.

In conclusion, the ED_50_ and ED_95_ of prophylactic bolus nitroglycerin in preventing the pituitrin-induced hypertension during laparoscopic myomectomy were 54 μg (95%CI: 35~66 μg) and 136 μg (95%CI:105~289 μg), respectively. Balancing the advantages and disadvantages, an initial dose of 100 μg of nitroglycerin appeared to offer optimal protection against pituitrin-induced hypertension yet avoid hypotensive episodes.

## Data availability statement

The raw data supporting the conclusions of this article will be made available by the authors, without undue reservation.

## Ethics statement

The studies involving human participants were reviewed and approved by the Ethics Committee of Jiaxing University Affiliated Women and Children’s Hospital. The patients/participants provided their written informed consent to participate in this study.

## Author contributions

JW, QX, and FX contributed to the conception and design of the study and wrote sections of the manuscript. JW organized the database and wrote the first draft of the manuscript. QX performed the statistical analysis. All authors contributed to the article and approved the submitted version.

## Conflict of interest

The authors declare that the research was conducted in the absence of any commercial or financial relationships that could be construed as a potential conflict of interest.

## Publisher’s note

All claims expressed in this article are solely those of the authors and do not necessarily represent those of their affiliated organizations, or those of the publisher, the editors and the reviewers. Any product that may be evaluated in this article, or claim that may be made by its manufacturer, is not guaranteed or endorsed by the publisher.
